# An integrated overview of the midgut bacterial flora composition of *Phlebotomus perniciosus*, a vector of zoonotic visceral leishmaniasis in the Western Mediterranean Basin

**DOI:** 10.1371/journal.pntd.0005484

**Published:** 2017-03-29

**Authors:** Wael Fraihi, Wasfi Fares, Pascale Perrin, Franck Dorkeld, Denis Sereno, Walid Barhoumi, Imed Sbissi, Saifedine Cherni, Ifhem Chelbi, Ravi Durvasula, Marcelo Ramalho-Ortigao, Maher Gtari, Elyes Zhioua

**Affiliations:** 1 Laboratory of Vector Ecology, Pasteur Institute of Tunis, Tunis, Tunisia; 2 Laboratory of Microorganisms and Active Biomolecules, University of Tunis-El Manar, Faculty of Sciences, Tunis, Tunisia; 3 MIVEGEC/Université de Montpellier CNRS/UMR 5244/IRD 224 - Centre IRD, Montpellier, France; 4 INRA - UMR 1062 CBGP (INRA, IRD, CIRAD), Montpellier SupAgro, Montferrier-Sur-Lez, France; 5 UMR177, Centre IRD de Montpellier, Montpellier, France; 6 Division of Infectious Diseases, Center for Global Health, Department of Internal Medicine, UNM School of Medicine Albuquerque, New Mexico, United States of America; 7 Department of Preventive Medicine and Biostatistics, Uniformed Services University of the Health Sciences (USUHS), Bethesda, Maryland, United States of America; Fundaçao Oswaldo Cruz, BRAZIL

## Abstract

**Background:**

The *Leishmania* developmental life cycle within its sand fly vector occurs exclusively in the lumen of the insect’s digestive tract in the presence of symbiotic bacteria. The composition of the gut microbiota and the factors that influence its composition are currently poorly understood. A set of factors, including the host and its environment, may influence this composition. It has been demonstrated that the insect gut microbiota influences the development of several human pathogens, such as *Plasmodium falciparum*. For sand flies and *Leishmania*, understanding the interactions between the parasite and the microbial environment of the vector midgut can provide new tools to control *Leishmania* transmission.

**Methodology/Principal findings:**

The midguts of female *Phlebotomus perniciosus* from laboratory colonies or from the field were collected during the months of July, September and October 2011 and dissected. The midguts were analyzed by culture-dependent and culture-independent methods. A total of 441 and 115 cultivable isolates were assigned to 30 and 11 phylotypes from field-collected and colonized *P*. *perniciosus*, respectively. Analysis of monthly variations in microbiota composition shows a species diversity decline in October, which is to the end of the *Leishmania infantum* transmission period. In parallel, a compilation and a meta-analysis of all available data concerning the microbiota of two Psychodidae genera, namely *Phlebotomus* and *Lutzomyia*, was performed and compared to *P*. *perniciosus*, data obtained herein. This integrated analysis did not reveal any substantial divergences between Old and New world sand flies with regards to the midgut bacterial phyla and genera diversity. But clearly, most bacterial species (>76%) are sparsely distributed between Phlebotominae species.

**Conclusion/Significance:**

Our results pinpoint the need for a more exhaustive understanding of the bacterial richness and abundance at the species level in Phlebotominae sand flies in order to capture the role of midgut bacteria during *Leishmania* development and transmission. The occurrence of *Bacillus subtilis* in *P*. *perniciosus* and at least two other sand fly species studied so far suggests that this bacterial species is a potential candidate for paratransgenic or biolological approaches for the control of sand fly populations in order to prevent *Leishmania* transmission.

## Author summary

The use of conventional microbiological methods gave us the opportunity to investigate the richness of symbiotic bacteria that inhabit the gut of *P*. *perniciosus* during its main period of activity. Our results were subsequently analyzed in the framework of what has been done on sand flies microbiota in order to validate our results and to address the question of the definition of the core bacterial microbiota of sand flies. A meta-analysis on the respective gut microbiota of Old and New World sand flies shows that the majority of bacterial species is observed only in one host whereas less than 8% are shared by more than two hosts. Our results pinpoint the need for a more exhaustive understanding of the microbiota composition and dynamic in phlebotominae, with the aim to implement new biological approaches for the control of sand fly populations in order to prevent *Leishmania* transmission.

## Introduction

Sand flies are vectors of various pathogens, including arboviruses and bacteria, but are best known as the principal vectors of *Leishmania*, the etiological agent of leishmaniasis, a neglected tropical disease with clinical symptoms varying in form from cutaneous to visceral [1,2]. According to the most recent reports, leishmaniasis affects nearly 12 million people located in tropical, subtropical, and Mediterranean regions [3,4] with an estimated 350 million people at risk [5]. Among all vector-borne diseases, visceral leishmaniasis (VL) is the second leading cause of death after malaria, with an annual incidence of 500,000 cases and 60,000 deaths each year [3,4]. To date, no effective vaccine is available against leishmaniasis, and treatments mainly rely on chemotherapy using pentavalent drugs. Currently, the effectiveness of the treatment varies because of adverse side effects on patients and the emergence of parasite drug resistance [6,7].

*Phlebotomus* and *Lutzomyia* are the main sand fly genera involved in the transmission of *Leishmania sp*. in both the Old and the New World [2, 8–10]. Sand flies become infected when they blood feed on an infected host. Ingested amastigote parasites undergo a complex developmental cycle within the sand fly and are limited to the midgut of the insect [11]. Thus, the midgut of the vector is the first point of contact between ingested parasites and the apical surface of the intestinal epithelial cells of the vector. Bacteria have been isolated from the midgut of *P*. *papatasi*, a vector of *Leishmania major*, the etiologic agent of zoonotic cutaneous leishmaniasis (ZCL) [12], and studies have suggested a role for these bacteria in the immune response and homeostasis [12–15]. Female sand flies feed on blood for egg laying. In addition to blood, they take sugar meals derived from a number of different sources, including leaves, fruit, and aphid honeydew. Such food sources offer many opportunities to ingest microorganisms [16–18]. The microbiota found in sand fly guts could mirror their diets.

In low- and middle-income countries, such as Tunisia, large vector eradication programs are challenging owing to limited resources. New approaches to control vector transmission of *Leishmania infantum* are of major interest. These programs are needed to control the transmission of *L*. *infantum* in Tunisia. Paratransgenesis has been suggested as a feasible strategy for controlling the transmission of pathogens by arthropod vectors. This approach consists of the use of genetically altered symbiotic bacteria that secrete effector molecules that kill the infectious agents. Since these bacteria should co-localize with the pathogen and be transmitted vertically to the next generation, they are introduced into vectors to block pathogen transmission [19–20]. This "Trojan-Horse" approach was initially developed to interfere with the transmission of *Trypanosoma cruzi* by its triatomine vector [19]. Among possible bacterial species that could be considered as candidates for the development of a paratransgenic approach, *Bacillus pumilus* and *Bacillus flexus* were identified as the most frequent cultivable bacteria identified in the midgut of *P*. *papatasi* field-collected from Tunisia, Turkey, and India [21]. In addition, *Bacillus subtilis* isolated from *Phlebotomus argentipes* is currently being considered as a possible candidate for paratransgenesis aimed at preventing *Leishmania donovani* transmission [22,23].

In North Africa, *Phlebotomus perniciosus* is the main vector of *L*. *infantum*, the etiologic agent of zoonotic visceral leishmaniasis (ZVL) [24]. We sought to develop a paratransgenic platform to control the transmission of *L*. *infantum* by *P*. *perniciosus*. Here, we assessed the richness of bacterial species of laboratory-reared and field-collected sand flies. We investigated the monthly variations of the bacterial diversity carried by sand flies in an endemic area of ZVL in Tunisia, during the period of *Leishmania infantum* transmission. We analyzed these new data within the context of previously published studies on the microbiota of sand flies.

## Materials and methods

### Sand fly collection, identification and gut dissection

Sand flies collection: Laboratory-reared *P*. *perniciosus* (Tunisian strain) was obtained from a colony maintained at the Vector Ecology Laboratory of Pasteur Institute of Tunis [25]. *Phlebotomus perniciosus* individuals were also collected in a sheep shelter in the village of Utique located in Northern Tunisia (37°08’N, 7°74’E), with the owner consent, by using CDC traps. Sand fly trapping was performed from dusk to dawn one night per month, from July to October 2011. This period corresponds to the period of main activity of *P*. *perniciosus* in Tunisia [26]. Field-collected sand flies were brought alive to the laboratory. However, as it is difficult to determine the age of field-collected sand flies, we arbitrarily attribute the day of their sampling as the day one. All field-collected sand flies were dissected within three days after collection. Laboratory-reared sand flies were dissected three-to-seven days after their emergence. Prior to dissection, each sand fly was rinsed in 70% ethanol for 3 minutes, followed by three successive rinsings in sterile PBS. Sand flies were then dissected on ice under stereo-microscope, in order to remove the midgut for bacterial identification and the genitalia for morphological identification to species level [26,27]. Only *P*. *perniciosus* females were used.

Gut dissection: Each sand fly gut was individually placed in 1.5 ml microcentrifuge tubes containing 200 μl of sterile PBS (pH 7.3), homogenized with a disposable pestle, and diluted from 10^−1^ to 10^−10^ in 200 μl PBS. Each homogenate was plated onto individual 1.5% agar plates with TSA (Trypticase Soy Agar), PCA (Plate Count Agar), YMA (Yeast Mannitol Agar) or Luedemann medium and incubated at 30°C for 2 to 4 days in aerobic conditions. Individual colonies were selected and used for further identification.

### DNA extraction

Chromosomal DNA extraction was performed as previously described [28]. After overnight incubation at 30°C in TSA, PCA, Luedemann or YMA medium, colonies were suspended in 500 μl of TE buffer (10 mM Tris-HCl, 0.1 mM EDTA, pH 8) to which 20 μl of lysozyme (35 mg/ml) was added and incubated at 37°C for 30 min. Then, 40 μl of sodium dodecyl sulfate (SDS 10%) and 5 μl of freshly prepared proteinase K (10 mg/ml) were added, and the solution was incubated at 30°C for 30 min. The solution was homogenized after the addition of 100 μl of 5 M NaCl and 80 μl of CTAB/NaCl (10%/0.7 M) and incubated at 65°C for 10 min. DNA was purified by the addition of phenol-chloroform-isoamyl alcohol (25:24:1, pH 8.0), followed by chloroform-isoamyl alcohol (24:1) and then precipitated by the addition of 0.6 volumes of isopropanol. DNA pellets were washed with 200 μl of 70% ethanol and dried at 37°C before being resuspended in TE buffer (10 mM Tris-HCl, 0.1 mM EDTA, pH 8) and stored at -20°C. Total DNA extraction for the Denaturing Gradient Gel Electrophoresis (DGGE) analysis was conducted on whole midguts dissected from sand flies using the same total DNA extraction protocol described above [28].

### Bacterial colony screening and identification

**Fig 1:**
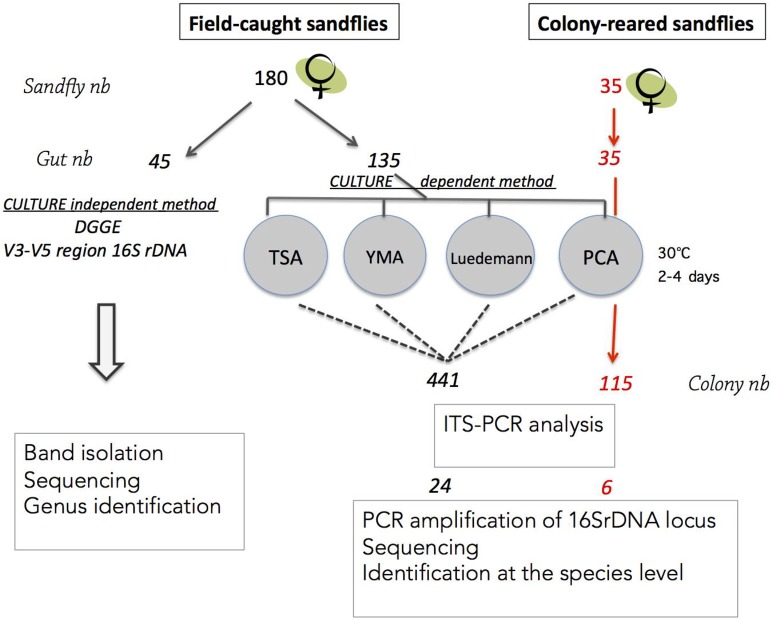
Schematic representation of the bacterial colonies isolation and identification procedure.

Fig 1 summarizes the procedure used for the isolation and identification of bacterial species. A total of 180 field-collected and 35 colonized *P*. *perniciosus* females were processed. From field-collected sand flies, 135 guts were used for culture-dependent identification and 45 guts were analyzed by DGGE, a culture-independent method. The 35 samples from colonized *P*. *perniciosus* were processed only for culture-dependent identification.

### Screening of colonies with ITS-PCR

The length and sequences polymorphisms of the Intergenic Transcribed Spacers (ITS), located between the 16S and 23S rRNA, is quite often due to the presence of tRNA genes. PCR amplification of the 16S-23S intergenic transcribed spacer regions between the rRNA genes (ITS) was performed for screening the bacterial phylotype diversity [29–31]. The universal primers, ITSF (5’-GTCGTAACAAGGTAGCCGTA-3’) and ITSR (5’-CAAGGCATCCACCGT-3’), are complementary to nucleotide (nt) positions 1423–1443 of the 16S rDNA and nt positions 38–23 of the 23S rDNA of *Escherichia coli*, respectively [30]. Each reaction tube contains 1X PCR buffer (Invitrogen), 2 mM MgCl_2_, 0.2 mM deoxynucleoside triphosphate mix, 0.1 μM of each primer, 0.5 U of Taq polymerase (Invitrogen) and 400 ng of DNA extracted from single colonies. The total volume was adjusted to 25 μl. Amplification parameters were as follows: initial denaturation at 94°C for 5 min, followed by 35 cycles at 94°C for 30 s, 50°C for 30 s, 72°C for 45 s, with a final extension step of 10 min at 72°C, using an ABS2720 thermocycler.

### Amplification of the 16S rDNA

Amplification of the 16S rDNA gene was carried out with universal primers SD-Bact-0008-a-S-20 and S-D-Bact-1495-a-S-20 [32]. Each reaction tube contained 1x PCR buffer (Invitrogen), 0.5 μM of each primer, 2.5 mM MgCl_2_, 200 ng of purified DNA, 0.2 mM dNTPs and 0.3 units of Taq polymerase (Invitrogen) and the total volume was adjusted to 25 μl. Samples were amplified according to the following cycle: an initial denaturation step at 94°C for 10 min, followed by 35 cycles at 94°C for 1 min, 55°C for 1 min, 72°C for 1 min and a final extension step of 10 min at 72°C, using an ABS2720 thermocycler. PCR amplicons were then purified using the QIAquick PCR Purification Kit (Qiagen) and sequenced.

### Denaturing-Gradient Gel Electrophoresis (DGGE) analysis

Amplification of the V3-V5 region of the 16S rDNA: PCR amplification targeting the 16S rDNA genes was performed using the universal primers specific to the bacterial domain: 907r (5’-CCGTCAATTCCTTTGATGTTT-3’) and 357f (5’-TACGGGAGGCAGCAG-3’) [33]. A 40-bp GC-clamp was added to primer 357f to avoid complete denaturation of the DNA and allow the separation of DNA strands during migration in denaturing conditions [34–36]. Each reaction tube contained 1x PCR buffer (Invitrogen), 2.5 mM MgCl_2_, 0.12 mM dNTPs, 0.3 mM of each primer, 1 U of Taq DNA polymerase (Invitrogen) and 50 ng of DNA in a final volume of 50 μl. Amplification parameters were as follows: an initial denaturation step at 94°C for 4 min, 10 cycles at 94°C for 30 s, 61°C for 1 min and 72°C for 1 min, followed by 20 cycles at 94°C for 30 s, 56°C for 1 min and 72°C for 1 min. At the end of these cycles, a final extension step was performed at 72°C for 10 min.

DGGE analysis: PCR products were run on a 7% polyacrylamide gel in a 40%–60% denaturing gradient of urea and formamide for 16S rDNA analysis. DGGE was performed using a BioRad DCode Universal Mutation Detection System at 100 V at 59°C for 17 hr, in 1.0 × TAE buffer (20 mmol/L Tris, 10 mmol/L acetate, 1 mmol/L EDTA pH 7.4). After electrophoresis, gels were stained for 30 min with ethidium bromide.

Identification of the DGGE Bands: Excised bands of DGGE gels were washed twice with 1 mL sterilized distilled water in a 1.5-mL tube. A portion of the gel piece (< 1 mm^3^) was used as the direct template for PCR to recover DNA fragments. Amplification conditions for the V3-V5 region were as follows: an initial denaturation step at 94°C for 4 min followed by 35 cycles at 94°C for 30 s, 56°C for 1 min and 72°C for 1 min and a final extension step at 72°C for 10 min. Primers were identical to those described above except that the forward primer had no GC-clamp attached. The amplified products were purified with the QIAquick PCR Purification Kit (Qiagen) and then sequenced.

### Sequencing of 16S rDNA

The 16S rDNA sequencing was carried out using the BigDye Terminator v3.1 Cycle sequencing Kit and the ABI 3130 sequence analyzer. The partial 16S rRNA gene sequences were compared with sequences available in the ribosomal database, release 11.4. Isolates were assigned at the species level on the basis of the 16S rRNA gene sequence similarity of the available sequences in the ribosomal database, measured by using the Seqmatch tool of RDP [37] (https://rdp.cme.msu.edu/). In addition, the partial 16S rDNA sequences were submitted to the BLASTn server of NCBI, using the 16S ribosomal RNA database (Bacteria and Archea) (http://blast.ncbi.nlm.nih.gov/Blast.cgi). The nucleotide similarity thresholds of the 16S rDNA sequences with the nearest neighbor were: ≥ 95% and 97.5% [38] applied at the genus and species levels, respectively.

### Diversity analysis

All the analyses were conducted with the R-vegan package, v. 2.0–10 [39]. α-diversity was calculated using Shannon’s and Simpson’s diversity indices. Correspondance analysis (CA analysis) on the monthly data was carried out with the FactomineR package (https://cran.r-project.org/web/packages/FactoMineR/) using the R language (http://www.R-project.org).

### Meta-analysis of Phlebotominae microbiota

All the published data concerning bacterial species identification associated with *Phlebotomus* and *Lutzomyia* species (the only two genera for which we have data) were compiled and analyzed. Studies describing the identification of the midgut bacteria at the family, class or phylum level were not considered. To assess bacterial richness associated with the adult sand fly, data were collected without taking into account the method of bacterial isolation (culture-dependent vs culture-independent) and identification (DNA sequencing of 16S rDNA, bacteriology). The overall dataset used in our analyses included ten Phlebotominae (*L*. *cruzi* [40], *L*. *longipalpis* [41,42], *L*. *evansi* [43], *P*. *argentipes* [22], *P*. *duboscqi* [44], *P*. *halepensis* [45], *P*. *papatasi* [21, 45–48], *P*. *sergenti* [45], *P*. *perfiliewi* [45], *P*. *chinensis* [49] and *P*. *perniciosus*) and their associated microbiota for the present study. Bacterial richness is visualized through network analysis using Cytoscape (http://www.cytoscape.org/) [50]. To achieve this goal, data were extracted from our own database (focused on Phlebotominae) as CSV files, containing vertices or nodes (representing hosts and bacteria) and edges (representing links). These files were loaded into Cytoscape v 3.4.0, a tool specializing in graphical representation. This graph was modified to keep only one edge between host and bacteria. Bacterial nodes were colored to show their degrees of interaction with hosts.

## Results

### Overall bacteria composition as revealed by culture-dependent and culture-independent analysis

**Fig 2:**
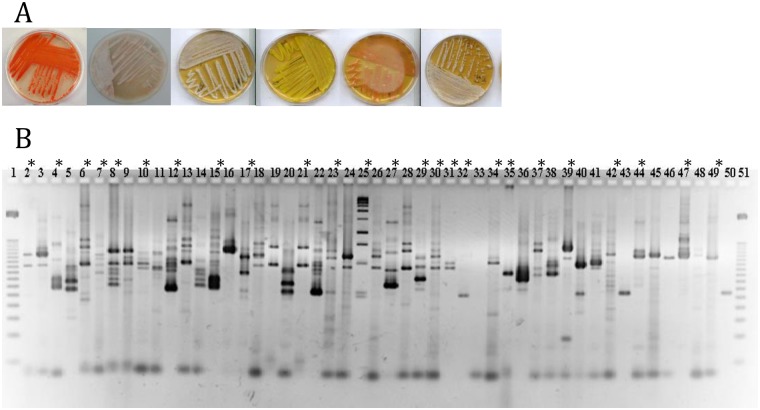
(A) Aspect of some gut isolated bacterial colonies grown on Luedemann medium. (B) Example of PCR-ITS analysis performed on 49 out of 556 bacterial colonies isolated from the guts of colony-reared and field-collected sandflies. * indicates the 25 individual ITS-PCR profiles whose colonies were further analyzed by 16S rDNA sequencing.

First, we determined the number of Colony Forming Units (CFU) of each individual midgut using PCA medium; they ranged from 5 to 121 per individual sand fly midgut. A total of 441 and 115 independent colonies were obtained from field-collected and colonized sand flies, respectively (Fig 1). Examples of the different types of bacterial colonies are shown in Fig 2A.

ITS-PCR analysis was used to dereplicate strain diversity among the 556 colonies. Each ITS profile is composed of one to five reproducible bands that display apparent molecular weights ranging from 50 to approximately 1,500 bp; these bands represent a phylotype (Fig 2B). Among the 441 colonies isolated from field-collected *P*. *perniciosus*, 25 distinct ITS-PCR profiles were identified (Fig 2B see *). Of a total of 115 independent colonies from colonized *P*. *perniciosus*, only 6 independent phylotypes were identified (Fig 2B). When possible, the 16S rDNA locus of a sample representative of each ITS-PCR profile was further amplified to attempt identification at the genus and species levels. All the bacterial colonies isolated from sand fly midguts belong to three phyla: Firmicutes, Actinobacteria, and Proteobacteria. For the field-collected *P*. *perniciosus*, the calculated midgut bacterial composition was: Firmicutes (53.5%), Actinobacteria (15.2%) and Proteobacteria (31.3%). For laboratory-reared sand flies, the midgut bacterial composition was Firmicutes (66.7%) and Proteobacteria (33.3%). We did not isolate bacteria belonging to the Actinobacteria phylum from the midgut of laboratory-reared sand flies. Nevertheless, only 35 females were processed and bacterial colonies were isolated solely using the PCA medium, which might have influenced the output of our analysis.

The results of isolating bacterial species from the midguts of field-collected and lab-reared *P*. *perniciosus*, performed in a culture dependent manner, are shown in Table 1. Of the six bacterial species identified in laboratory-reared sand flies (Table 1), three are also found in the midgut of field-collected sand flies (*Stenotrophomonas maltophilia*, *Bacillus sp*., *Lysinibacillus sp*.) (Table 1). We isolated *Veillonella* sp. and *Burkholderia fungorum* only from the laboratory-reared sand flies (Table 1). Overall, the bacterial richness recorded in field-collected sand flies, at the species level, seems to be more important than in laboratory-reared flies, even if the total number of lab-reared flies studied is small.

**Table 1:** Bacterial species assignation.

*P*. *perniciosus* origin	Medium	Species assignation	Similarity %	Accession N°	Length (bp)	Phylum
Wild	Luedemann	*Ochrobactrum intermedium*	99	KY303725	879	α-proteobacteria
Wild	Luedemann	*Sporosarcina koreensis*	98	KY303698	830	Firmicutes
Wild	Luedemann	*Ochrobactrum* sp.	98	KY303699	879	α-proteobacteria
Wild	Luedemann	*Rhizobium pusense*	97	KY303727	902	α-proteobacteria
Wild	Luedemann/PCA	*Enterococcus faecalis*	99	KY303701	908	Firmicutes
Wild	Luedemann	*Roseomonas ludipueritiae*	99	KY303702	843	α-proteobacteria
Wild	Luedemann	*Kocuria polaris*	98	KY303703	786	Actinobacteria
Wild	Luedemann	*Nocardia ignorata*	99	KY303705	683	Actinobacteria
Wild/Colony	Luedemann/PCA	*Lysinibacillus sp*.	98	KY303706	751	Firmicutes
Wild	PCA	*Bacillus oleronius*	99	KY303707	838	Firmicutes
Wild/Colony	PCA	*Bacillus* sp.	99	KY303708	836	Firmicutes
Wild	PCA	*Bacillus subtilis*	98	KY303709	836	Firmicutes
Wild	PCA	*Bordetella avium*	98	KY303726	527	β-proteobacteria
Wild	PCA	*Brevundimonas terrae*	98	KY303710	835	α-proteobacteria
Wild	PCA	*Staphyloccocus epidermidis*	98	KY303711	958	Firmicutes
Wild	PCA	*Bacillus galactosidilyticus*	98	KY303712	918	Firmicutes
Wild/Colony	PCA	*Stenotrophomonas maltophilia*	98	KY303713	910	γ-proteobacteria
Wild	PCA	*Serratia* sp.	99	KY303714	800	Firmicutes
Colony	PCA	*Bacillus casmanesis*	97	KY303715	981	Firmicutes
Colony	PCA	*Burkholderia fungotum*	98	KY303716	912	β-proteobacteria
Colony	PCA	*Veillonella* sp.	97	KY303717	599	Firmicutes
Wild	YMA	*Microbacterium* sp.	98	KY303718	1002	Actinobacteria
Wild	YMA	*Saccharomonospora* sp.	98	KY303719	912	Actinobacteria
Wild	YMA	*Micrococcus* sp.	99	KY303720	948	Actinobacteria

**Fig 3:**
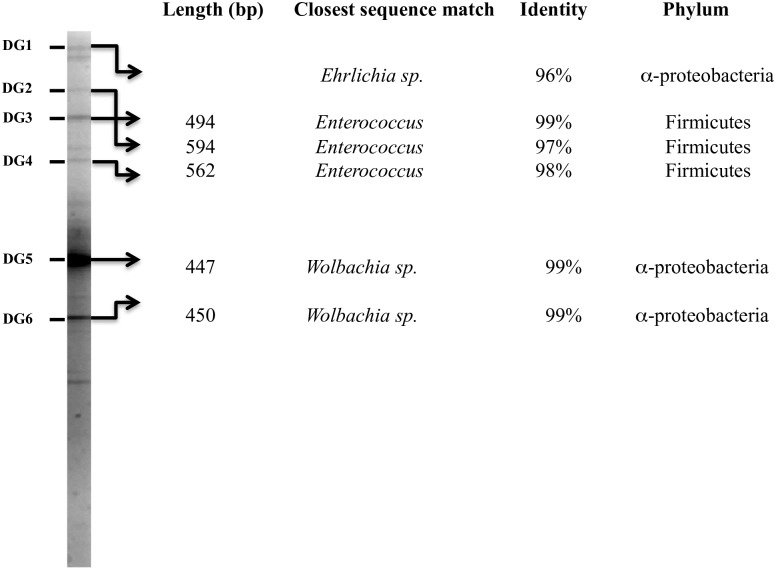
DGGE profile of amplified gene fragments of bacterial 16S rDNA from midguts of *P*. *perniciosus* on polyacrylamide gel (7%) and band identification for subsequent sequence analysis.

To further characterize the bacterial richness in field-collected sand flies, a culture-independent method (DGGE) was performed on the 45 dissected midguts (Fig 1). Despite variation in the number and intensity of the bands detected, the observed DGGE profile is composed of at least 12 distinguishable bands. Among these bands, six were successfully sequenced. In addition to bacteria already identified using culture-dependent methods, like *Enterococcus* sp. (Accession N° KY303721 and KY303722), we also identified *Wolbachia* sp. and *Ehrlichia* sp. (Fig 3). BLASTing the sequence from the DG5 band (459 bp Accession N° KY303723) indicated an overall similarity of 99% with the Pel strain of *Wolbachia*, isolated from *Culex quinquefasciatus* (NR-074127.1). The same query on the RDP database disclosed 98% similarity with *Wolbachia inokumae* DQ402518, which was already found in field collected *P*. *perniciosus* from Marseille, France [51]. A search in the RDP database with the sequence obtained from the DG1 band (718 bp, Accession N° KY322518) produced hits with various species of *Ehrlichia*, including 96% similarity with *Ehrlichia canis*-M73226. A similarity of 96% with *Ehrlichia ewingii* (NR-044747) was found when BLAST analysis was performed on the 718-bp DNA fragment (Fig 3). To our knowledge, this is the first report of the presence of *Ehrlichia sp*. DNA in sand fly midguts.

### Sand fly-associated bacteria, as revealed via meta-analysis of the literature data

**Fig 4:**
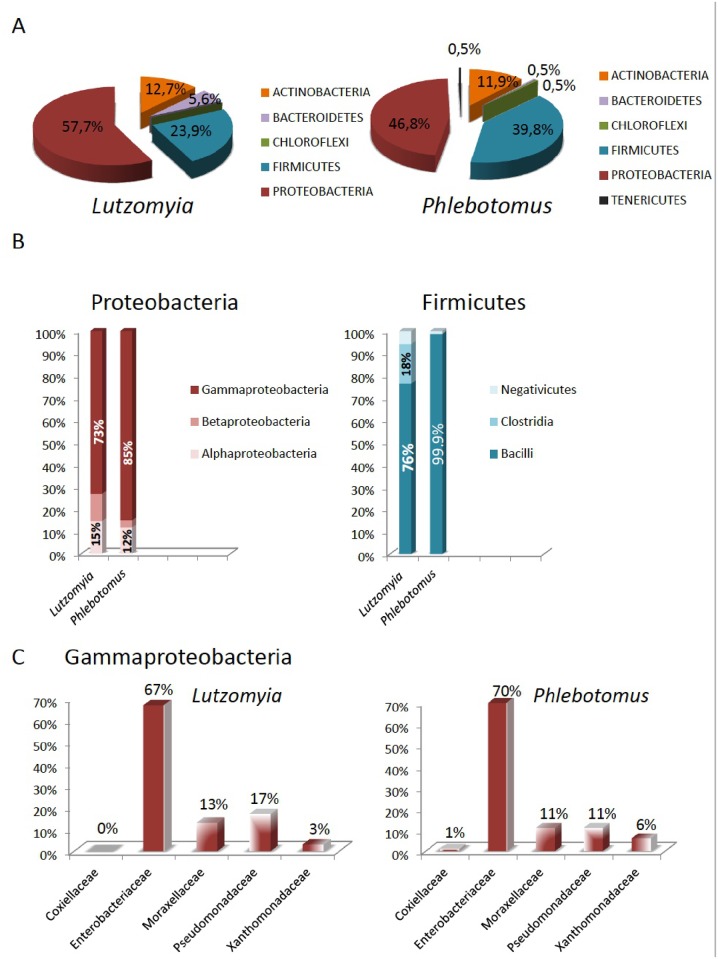
(A) Composition of the bacterial phyla found in *Lutzomyia* genus and *Phlebotomus* genus sand flies. Pie charts show the proportion of each of the five phyla detected namely Proteobacteria, Firmicutes, Actinobacteria, Bacteroidetes and Chloroflexi. (B) Relative proportion of Proteobacterial classes observed in *Lutzomyia* and *Phlebotomus* genera sand flies, respectively on the left and relative proportion of Firmicutes bacterial classes found in *Lutzomyia* and *Phlebotomus* genera respectively on the right. (C) Relative proportion of the major families of bacteria in the gammaproteobacterial class: “Enterobacteriales” order (Enterobacteriaceae), the Pseudomonadales order (Moraxellaceae and Pseudomonadaceae), the Xanthomonadales order (Xanthomonadaceae) and the Legionellales order (Coxiellaceae) in *Lutzomyia* (right panel) and *Phlebotomus* (left panel).

A meta-analysis was conducted to assess the bacterial species diversity of *Phlebotomus* and *Lutzomyia* microbiota. This analysis included previously published studies concerning adults of seven phlebotomine sand fly species (*P*. *argentipes*, *P*. *chinensis*, *P*. *duboscqi*, *P*. *halepensis*, *P*. *sergenti*, *P*. *papatasi*, *P*. *perfiliewi*) our study reported on *P*. *perniciosus* and previously published data reported on three *Lutzomyia* species (*L*. *cruzi*, *L*. *evansi*, *L*. *longipalpis*) [22,40–49]. Owing to the small number of studies conducted on the microbiota of Phlebotominae and the lack of information about sex in several cases, we chose to not take into account the genera of the specimen in order to highlight trends. This analysis shows that most bacteria identified from Old World sand fly species belong to the Firmicutes phylum, 39,8% (Fig 4A left panel) (41–42% for our study on *P*. *perniciosus*) and the Proteobacteria phylum, 46,8% (Fig 4A right panel) (37% for our study on *P*. *perniciosus*). Bacteria of the *Bacteroides* genus are not recorded in the present study and represent only 0.5% calculated from the pooled published data (i.e., Meta-set). Bacteria of the Actinobacteria phylum account for 11.9% of the Meta-set (20%, in our study on *P*. *perniciosus*). In *Lutzomyia sp*., more than 57% of bacteria currently characterized, belong to the Proteobacteria phylum (Gram-negative bacteria), Firmicutes representing 23.9% and Actinobacteria 5.6%. Bacteria of the Bacteroidetes phylum account for approximately 6% of the species in *Lutzomyia* but only 0.5% in Old World sand fly species (Fig 4A). Nevertheless, we did not notice significant differences in Bacterial phylum composition between Old World and New World sand flies (chi-squared = 5.8226, df = 2, p-value = 0.0544) (Fig 4A).

Within the Proteobacteria phylum, compared with the alpha-, beta- and deltaproteobacteria identified, gammaproteobacteria are by far the most frequently found bacterial class in *Lutzomyia* and *Phlebotomus* species (Fig 4B right panel). Within the Firmicutes phylum, a higher number of classes is observed in *Lutzomyia*, with bacteria belonging to *Negativicutes*, *Bacilli*, and *Clostridia*. The *Bacilli* class is almost the sole representative of Firmicutes class in the Old World sand fly species (Fig 4B left panel).

In the Gammaproteobacteria class, bacterial species of the *Enterobacteriaceae* family are the most represented (more than 60% so far isolated) in both the Old and New World sand fly species, followed by bacteria belonging to the *Pseudomonadaceae* and *Moraxellaceae* families (less than 20%) and *Xanthomonadaceae*, with less than 10% (Fig 4C right and left panel). Bacteria of the *Coxiellaceae* family have only been isolated from Old World sand fly species.

**Fig 5:**
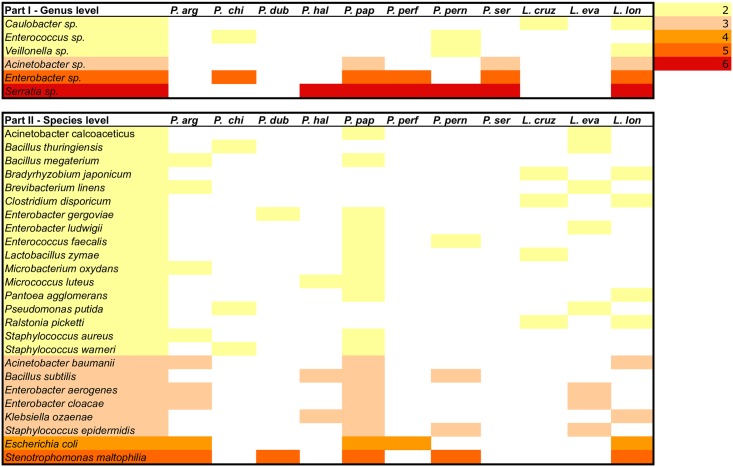
Bacterial genera (part I) and species (part II) shared by two or more of the eleven Phlebotominae sand flies species. Color chart indicate the occurrence of the bacterial genera or species.

Our meta-analysis shows that bacteria of the *Serratia* genus has been identified in almost all Old World and New World sand fly species so far studied, but *Serratia marcescens* was characterized only in *P*. *duboscqi*. Bacteria of the *Enterobacter* genus are found in five of the eleven sand fly species studied. *Enterobacter cloacae* and *Enterobacter aerogenes* were recorded in three sand fly species, while *Enterobacter gergoviae* and *Enterobacter ludwigii* were found in two sand fly species. The most frequently isolated bacteria in sand flies are *Stenotrophomonas maltophilia* (Pseudomonadaceae), followed by *Escherichia coli* (Enterobacteriaceae), *Klebsiella ozaenae* (Enterobacteriaceae), and *Staphylococcus epidermidis* (Staphylococcaceae). *Bacillus subtilis* (Bacillaceae) and *Acinetobacter baumannii* (Moraxellaceae) were identified in three of the eleven sand fly species currently studied (Fig 5).

**Fig 6:**
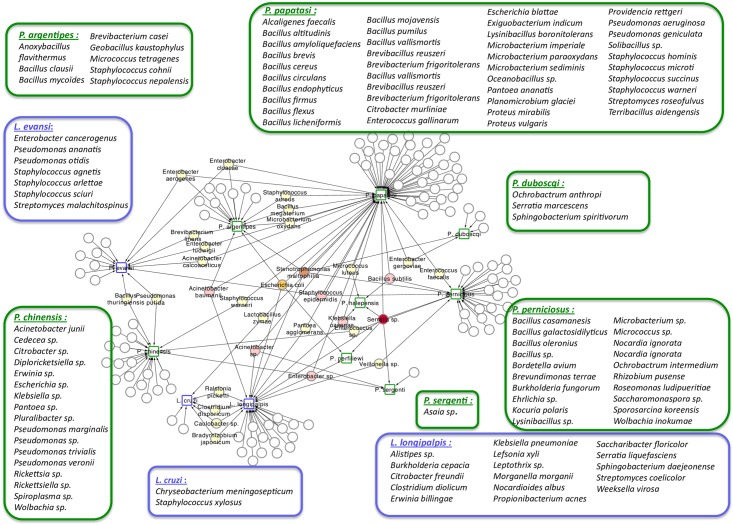
Network analysis showing the shared bacteria species found in *Phlebotomus* sand flies including *P*. *argentipes*, *P*. *duboscqii*, *P*. *halepensis*, *P*. *papatasi*, *P*. *perlifiliewi*, *P*. *perniciosus*, *P*. *sergenti*, *and P*. *chinensis*) identified by squares surrounded by green and bacteria found in *Lutzomyia* sand flies including *L*. *evansi*, *L*. *cruzi* and *L*. *longipalpis* identified with squares surrounded by blue. Shared bacteria are identified by the same colours used in Fig 5.

Despite that neither statistical nor bioinformatics analysis were performed to test the existence of biological patterns between sand fly species and their corresponding microbiota, the network representation displayed in Fig 6 suggests some relationships between the eleven studied New World and Old World sand fly species and the bacteria inhabiting their guts. As an example, the *Bacillus* genus is found in almost all Old World sand fly species. *Bacillus subtilis* was isolated from *P*. *halepensis*, *P*. *papatasi* and *P*. *perniciosus*. *Bacillus megaterium* was isolated from *P*. *papatasi* and *P*. *argentipes*. *Bacillus oleronius*, *Bacillus brevis*, *Bacillus endophyticus*, *Bacillus pumilus*, *Bacillus circulans*, *Bacillus mojavensis*, *Bacillus firmus*, *Bacillus licheniformis*, *Bacillus vallismortis*, *Bacillus cereus*, *Bacillus amyloliquefasciens*, *Bacillus altitudinus* and *Bacillus flexus* were isolated only from *P*. *papatasi*. *Bacillus closei* and *Bacillus mycoïdes* were isolated only from *P*. *argentipes*. *Bacillus oleronius*, *Bacillus galactosidilyticus*, and *Bacillus casamensis* were isolated only from *P*. *perniciosus* (Fig 6). *Bacillus thuringiensis* is the only species of the *Bacillus* genus that was isolated from *L*. *evansi* and *P*. *chinensis*, two sand fly species belonging to the New World and Old World, respectively (Fig 6).

**Fig 7:**
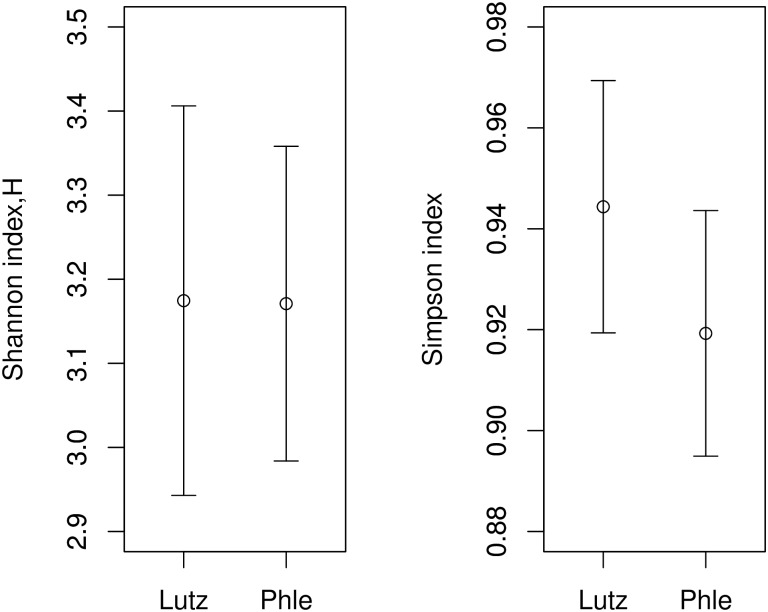
Shannon’s diversity (left side) and Simpson’s diversity values (right side) for *Lutzomyia* (Lutz) and *Phlebotomus* (Phle). Error bars are 95% confidence interval.

Nevertheless, in the Meta-Set no significant differences in the microbiota composition at the genus level was observed as demonstrated in the Fig 7 that depicts the Shannon (left) and Simpson (right) indices of diversity for Old World (i.e *Phlebotomus*) and New World sand flies (*Lutzomyia*).

### Monthly dynamics of the gut bacterial consortium during the main period of *P*. *perniciosus* activity

**Fig 8:**
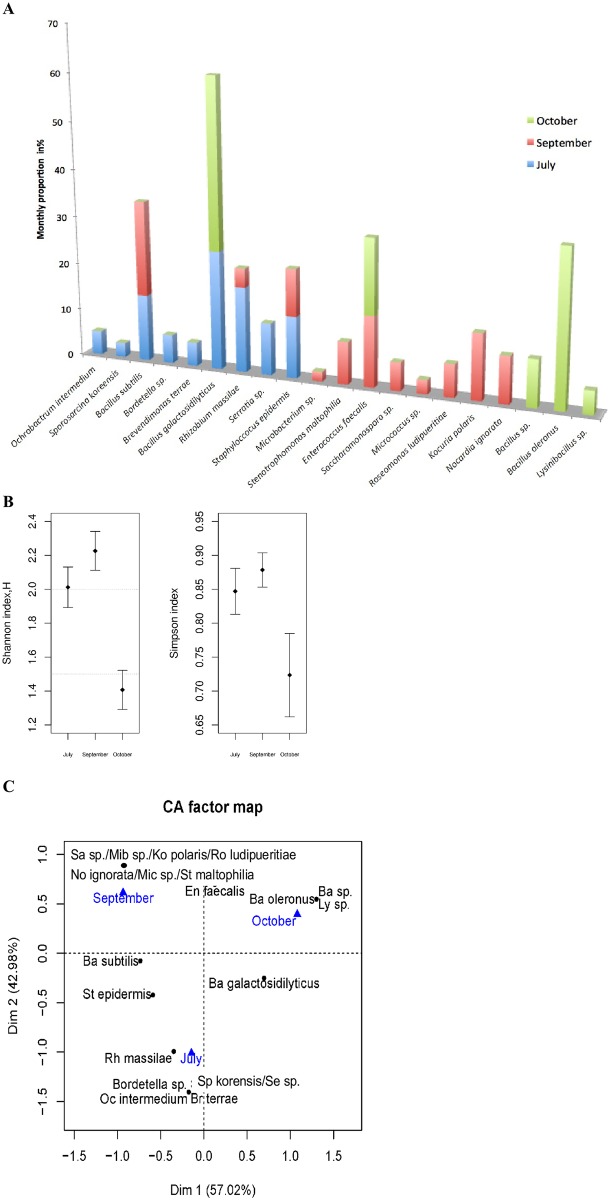
(A) Dynamic of the midgut bacterial richness and abundance during July, September, and October 2011. An ITS-PCR profile was assigned to a bacterial species and identified by the sequencing of the 16S rDNA locus. The percentage of each bacterial species was obtained by counting the number of colonies having the same ITS-PCR profile and expressed as a percentage of the total colonies isolated during the considered month. (B) Shannon’s diversity value (left side) and Simpson’s diversity value (right side) for the months of July, September and October respectively. Error bars are 95% confidence interval. (C) Correspondance analysis (CA) based on bacterial species frequencies (in black) according the different months (July, September and October in blue). Bacteria are identified by an abbreviation: Ba, *Bacillus*; Br, *Brevundimonas*; En, *Enterococcus*, Ko, *Kocuria*; Ly, *Lysinibacillus*; Mib, *Microbacterium*; Mic, *Micrococcus*; Oc, *Ochrobactrum*; No, *Nocardia*; Rh, *Rhizobium*; Ro, *Roseomonas*; Sa, *Sacharomonospora*; Se, *Serratia*; Sp, *Sporosarcina*; Ste, *Stenotrophomonas; St*, *Staphylococcus*.

Fig 8A depicts the monthly proportions of each previously characterized bacterial species. To perform this analysis, bacterial species identification was linked to each individual phylotype recorded by PCR-ITS analysis. Then, the number of colonies harboring the same ITS-PCR profile was determined and their monthly proportion calculated.

In July, among the nine bacterial species identified in midguts of wild *P*. *perniciosus*, only bacteria belonging to two phyla, Proteobacteria and Firmicutes, were found. In September, the bacteria belonged to the Actinobacteria, Proteobacteria and Firmicutes phyla. In October, only bacteria belonging to Firmicutes were isolated (Fig 8A). Shannon and Simpson indices of diversity confirmed a lower diversity in October (Fig 8B). Correspondence analysis performed on data from monthly dynamics highlights the contrasting situation between October and September/July, depicted on the first axis. October is characterized by *B*. *oleronus* and unidentified *Bacillus* and *Lysinibacillus* species. The differences between July and September/October is depicted by the second axis of the correspondance analysis (Fig 8C). This analysis further reinforces our observation of a monthly evolution of the microbiota within the sand fly gut.

## Discussion

Currently, there are considerable efforts to study arthropod gut microbiota, especially those of medically important vectors. The microbiota is considered in the context of possible extended phenotypes conferred on the insect hosts that allow niche diversification and rapid evolution [52]. As early as 1929, Adler and Theodor [53] suggested that the presence of microorganisms in the guts of sand flies might impact the development of the parasite *Leishmania*. In the mid-80s, the team of Shlein and collaborators [12] observed a large number of “germ (bacteria) contaminations” in guts of wild-caught female *P*. *papatasi*. However, the composition of the sand fly’s gut microbiota was studied much later by Dillon *et al*. [54]. *Ochrobactrum sp*. was the first bacterium to be isolated from the midguts of *P*. *duboscqi*, a proven vector of *L*. *major* in Sub-Saharan Africa [44], and from other sand fly species [40], including laboratory-reared *Lutzomyia longipalpis* [55] and New World *L*. *intermedia* [56]. This bacterium, probably ingested by larva, passes to nymphs and up to the adults through transstadial transmission [44]. Recently, several publications were dedicated to the study of the microbial composition associated with the digestive tract of sand flies. Only a few studies concerning biotic and abiotic factors influencing the composition of the bacterial community of the midgut of sand flies were performed.

This study brings additional evidence on the microbiota composition in the midgut of *P*. *perniciosus*. Our results suggest that lab-reared *P*. *perniciosus* display a lower bacterial richness in their midgut than in field-collected sand flies. This difference is likely due in part to the type of food diet ingested by larvae and adults during rearing. In the laboratory, *P*. *perniciosus* larvae are fed sterile chaw (50% rabbit food plus 50% rabbit feces). After emergence, glucose is the main source of carbohydrates for adults [25]. Under natural conditions, larvae, as well as adult *P*. *perniciosus*, have a wide variety of diet including various sources of blood meals [18,57]. Therefore, the nature of the feeding regimen leads to a striking contrast between field-collected and laboratory-reared sand flies, which might explain the lower bacterial richness observed in colonized sand flies.

Among the bacterial genera found associated with *P*. *perniciosus* midgut, we identified isolates belonging to the *Burkholderia* genus and *Stenotrophomonas maltophilia*, an aerobic non-fermentative and a Gram-negative bacterium. We also identified bacterial species commonly found in the digestive tract of humans or other mammals, but which have not yet been described in the midguts of sand flies, like *Veillonella sp*. In addition *Sporosarcina koreensis*, *Rhizobium pusense* and *Nocardia* (a rare endophyte bacterium) have never been found in association with the sand fly gut. The richness of sand fly-associated bacteria, illustrated by the meta-analysis, point to some interesting outcomes. In *Lutzomyia sp*., more than 57% of identified bacteria belong to the Proteobacteria phylum (Gram-negative bacteria), whereas for Old World sand fly species, including *P*. *perniciosus*, Proteobacteria (47%) and Firmicutes (40%) are preponderant. Such a difference in the gut microbiota composition might be due to a number of factors, including the long divergence of evolution between the two subgenera [2]; some new studies are required to assess this observation. Another surprising finding is the high richness of *Bacillus* species found in Old World sand flies, in which the majority of these bacteria are host specific (Fig 6). *Stenotrophomonas maltophilia*, that has emerged as an important opportunistic pathogen [58] was found to inhabit the gut of most of the sand fly species so far studied. This bacterial species is a common microorganism found in aqueous habitats, plant rhizosphere, animal food and water sources. Thus, delineating the origin of the colonization of midguts by *S*. *maltophilia* and evaluating its role, if any, in the sand fly biology and physiology are of major importance.

Our results have, for the first time, disclosed monthly variation in the diversity of the sand fly’s gut microbiota, during the period of transmission of *L*. *infantum*. In fact, it appears that the richness of the gut microbiota is related to sand fly seasonal activity. This diversity could reflect the environmental conditions, such as temperature and humidity, but it may also be linked to variations in plant cover, such as flower blooming. At the beginning of the sand fly season (July), *Ochrobactrum* sp. and *Serratia* sp., both affiliated with the Proteobacterium phylum, were the principal bacterial genera isolated. The peak of activity of *P*. *perniciosus* occurs in September and October, a period that also corresponds to the *L*. *infantum* transmission season [59]. The analysis of the gut bacterial flora of sand flies collected in September reveals a higher diversity (Fig 8). In particular, we recorded the presence of *Microbacterium*, *Micrococcus*, *Kocuria*, *Stenotrophomonas*, and *Bacillus sp*. (Actinobacteria, Proteobacteria and Firmicutes). In July, *O*. *intermedium* and *Serratia sp*. are the dominant bacteria genera in the midgut of *P*. *perniciosus* and these bacteria became undetectable towards the main peak of sand fly activity identified in Tunisia, i.e., during the months of September and October [59]. The prevalence of *L*. *infantum* infection in the *P*. *perniciosus* population increases over the summer months and reaches a peak of 9% during September-October [60,61]. *Ochrobactrum intermedium* has been found previously to negatively affect *Leishmania mexicana* infection in *L*. *longipalpis* [55]. Certain strains of *S*. *marcescens* are capable of producing a pigment called prodigiosin, which ranges in color from dark red to pale pink depending on the age of the colonies. Derivatives of prodigiosin have recently been found to have anti-*T*. *cruzi* and anti-*Leishmania* (*Leishmania mexicana*) activity by promoting mitochondrial dysfunction leading to parasite programmed cell death [62,63]. To what extent such interplay between the bacterial colonies that exert toxic effects might interfere with the dynamic of *L*. *infantum* transmission awaits further investigation.

Sand flies are vectors of medical and veterinary importance. Understanding the establishment of the sand fly microbiota is critical towards clarifying underlying details of sand fly *Leishmania*-microbiota interactions [64]. Bacteria such as *O*. *intermedium*, which has been previously characterized in the guts of larvae, pupae, and adults of *P*. *duboscqi* [44], is an opportunistic pathogen to humans [65]. *Serratia sp*., an entomopathogenic bacteria found in this study, has been previously isolated from *L*. *longipalpis* [40] and *L*. *intermedia* [56]. *Bordetella avium*, isolated only once from a specimen caught during July, has never been previously isolated from sand fly midgut microflora. *Bordetella avium* is a highly pathogenic bacterium, causing the avian bordetellosis [66]. *Klebsiella ozaenae*, known also as a human pathogenic bacterium, has been found in four out of the ten studied sand fly species (not isolated in this study). *K*. *ozaenae* was isolated from the midgut of gravid and freshly fed females of *P*. *papatasi* and *P*. *halepensis* [45] and from some *Lutzomyia* species (Fig 5). *Klebsiella* species are ubiquitous in nature [67,68] and are recorded in all habitats where sand flies proliferate. Moreover, the presence of *K*. *ozaenae* in the midgut of gravid females [45] will highlight their capacity to survive in the gut of this insect. Nevertheless, as for all bacterial species known to be etiological agents of human diseases, the sole observation of their presence in sand fly gut is not sufficient to incriminate sand flies as a potential vector but gives information on the bacterial dissemination via blood-feeding insects. The data collected are not sufficient to incriminate sand flies as a biological vector of *K*. *ozaenae* but are enough to raise suspicion regarding their role in the dissemination of *K*. *ozaenae*. Furthermore, whether certain clinical outcomes from leishmaniasis may be linked to bacteria potentially deposited during the *Leishmania*-infected sand fly bite still remains to be fully investigated [68]. These studies will not only shed light on the effect of the gut bacterial community on the sand fly fitness but also on the establishment and the transmission of *Leishmania* parasites in endemic areas.

This meta-analysis aimed to identify the best bacterial candidate for a paratransgenic approach. Our study is based on data aggregated from various publications that use culture-dependent and culture-independent methodologies and various set of technical approaches used to study the sand fly microbiota. For these reasons, conclusions raised with this study should be taken with caution and analyzed in the light of the limitations and pitfalls inherently associated with the compilation of heterogeneous data. Among limitations, some are linked to the physiological state of the sample. The gut microbiome is highly dynamic [69] and therefore influences the outcome of the analysis. When using a culture-dependent approach, we have to keep in mind that only 20% of environmental bacteria can be grown on a growth medium [70]. Therefore, the composition of the microbiota is not a direct reflection of the bacterial community structure (abundance and richness) inside the insect, but an altered version of the ecosystem from where they came. Nucleic acid-based analysis, involving historically used methods (such as construction and Sanger sequencing of metagenomic clone libraries, automated ribosomal internal transcribed spacer analysis (ARISA), terminal restriction fragment length polymorphism (T-RFLP), denaturing gradient gel electrophoresis (DGGE)) and next generation sequencing technology require a critical step that must combine an efficient cell disruption without DNA degradation and uniform nucleic acid extraction. Unfortunately, no consensus protocol for microbial DNA extraction of insect-associated microbiota is currently available [70]. Although 16S rRNA gene sequencing is highly useful with regards to bacterial classification, it has a low phylogenetic power at the species level for some genera [71,72]. Depending on the 16S rRNA variable region targeted and the database used to perform the taxonomic profiling, misassignation of bacterial OTU at the species level could be frequent [73]. Nevertheless, taking into account all the above mentioned limits and pitfalls, we think that an exhaustive approach aimed at collecting a maximum of data on the microbiota of sand flies will give key information on the most commonly identified bacteria in sand fly species and those that are more specific.

Our groups are interested in the development of a paratransgenic platform to control the transmission of leishmaniasis. To that end, a strain of the non-pathogenic *Bacillus* species (*Bacillus subtilis*), isolated from *P*. *papatasi*, is proposed as a possible candidate for paratransgenic approach. In this study, we isolated *B*. *subtilis* from *P*. *perniciosus* midgut, in addition to other *Bacillus* species (*Bacillus oleronius*, *Bacillus casamensis*, *Bacillus galactosidilyticus* and *Bacillus* sp.*)*. Bacteria belonging to the *Bacillus* genus seem to display a host-specific distribution, with only *B*. *subtilis* being isolated in more than one sand fly species (*P*. *halepensis*, *P*. *papatasi*, and *P*. *perniciosus*). In addition, we observed that no bacteria belonging to the *Bacillus* genus have been characterized to date in adult New World sand fly species. Therefore, even if this bacterium possesses the main advantages of being non-pathogenic, easy to cultivate and to perform genetic manipulation, its use for paratransgenic control of *Leishmania* can be challenged by its capacity to establish long-term colonies in the gut of various sand fly species. In particular, if a paratransgenic approach is developed using *B*. *subtilis* as a host, it will be essential to probe its capacity to efficiently colonize the gut of *Lutzomyia* species and of other Old World sand fly species in which this bacterium has yet not been found in the gut. Thus, it will be of major epidemiological importance to develop a regional strategy for each endemic area with different bacterial isolates.

## Conclusion

The knowledge of interactions between sand flies, *Leishmania* and nonpathogenic microorganisms that inhabit the gut will help to delineate an appropriate bacterial host recipient that can be used for paratransgenesis designed to prevent *Leishmania* transmission. The identification at the species level of the midgut’s cultured flora of *P*. *perniciosus*, linked to its seasonal variation, is likely to provide new perspectives towards a better understanding of the role of the gut bacterial community on sand fly-pathogen interactions. This knowledge is crucial in order to implement control strategies for sand fly zoonotic visceral leishmaniasis.

## References

[1] Maroli M, Feliciangeli MD, Bichau L, Charrel RN, Gradoni L: Phlebotomine sandflies and the spreading of leishmaniases and other diseases of public health concern. Med Vet Entomol. 2013; 27:123–47. 10.1111/j.1365-2915.2012.01034.x 22924419

[2] Akhoundi M, Kuhls K, Cannet A, Votýpka J, Marty P, Delaunay P, Sereno D. A historical overview of the classification, evolution, and dispersion of *Leishmania* Parasites and Sandflies. PLoS Negl Trop Dis. 2016; 10:e0004349 10.1371/journal.pntd.0004349 26937644 PMC4777430

[3] Desjeux P. Leishmaniasis: current situation and new perspectives. Comp Immunol Microbiol Infect Dis. 2004; 27:305–18. 10.1016/j.cimid.2004.03.004 15225981

[4] Alvar J, Vélez ID, Bern C, Herrero M, Desjeux P, Cano J, Janin J, WHO Leishmaniasis control team. Leishmaniasis worldwide and global estimates of its incidence. PLoS ONE. 2012; 7:e35671 10.1371/journal.pone.0035671 22693548 PMC3365071

[5] World Health Organization (WHO). Urbanization: An increasing risk factor for Leishmaniasis. Wkly Epidemiol Record. 2002; 77:365–72. 12428426

[6] Seblova V, Oury B, Eddaikra N, Aït-Oudhia K, Pratlong F, Gazanion E, Maïa C, Volf P, Sereno D. Transmission potential of antimony-resistant *Leishmania* field isolates. Antimicrob Agents Chemother. 2014; 58:6273–76. 10.1128/AAC.02406-13 25049256 PMC4187949

[7] Sereno D, Maia C, Aït-Oudhia K. Antimony resistance and environment: Elusive links to explore during *Leishmania* life cycle. Int J Parasitol Drugs Drug Resist. 2012; 2:200–03. 10.1016/j.ijpddr.2012.07.003 24533281 PMC3862447

[8] Depaquit J, Ferté H, Leger N. The subgenus *Paraphlebotomus (Phlebotomus*, *Phlebotominae*, *Psychodidae*, *Diptera)*: a review. Morphological and Molecular Studies. Ann Pharm Fr. 2002; 58:333–40. 11060410

[9] Dedet JP. Les leishmanioses en Afrique du Nord. Bull Inst Pasteur. 1979; 77:85–94.

[10] Ready PD. Biology of phlebotomine sand flies as vectors of disease agents. Annu Rev Entomol. 2013; 58:227–50. 10.1146/annurev-ento-120811-153557 23317043

[11] Rogers ME, Hajmova M, Joshi MB, Sadlova J, Dwyer DM, Volf P, Bates PA. *Leishmania* chitinase facilitates colonization of sand fly vectors and enhances transmission to mice. Cell Microbiol. 2008; 10:1363–72. 10.1111/j.1462-5822.2008.01132.x 18284631 PMC2408650

[12] Schlein Y, Polacheck I, Yuval B. Mycoses, bacterial infection an antibacterial activity in sandflies (*Psychodidae*) and their possible role in the transmission of leishmaniasis. Parasitol. 1985, 90:57–66. 10.1017/s0031182000049015 3982854

[13] Dillon RJ, El Kordy E, Lanee RP. The prevalence of a microbiota in the digestive tract of *Phlebotomus papatasi*. Ann Trop Med Parasitol. 1996; 90:669–73. 9039284 10.1080/00034983.1996.11813102

[14] Tanada Y, Kaya HK. Associations between insects and nonpathogenics microorganisms In Tanada Y. & Kaya H.K. (eds.), Insect pathology. Academic Press, New York; 1993 pp.12–51.

[15] Dillon RJ, Dillon VM. The gut bacteria of insects: nonpathogenic interactions. Annu Rev Entomol. 2004; 49:71–92. 10.1146/annurev.ento.49.061802.123416 14651457

[16] Cameron MM, Pessoa FA, Vasconcelos AW, Ward RD. Sugar meal sources for the phlebotomine sandflies *Lutzomyia longipalpis* in Ceara State, Brazil. Med Vet Entomol. 1995; 9:263–72. 7548943 10.1111/j.1365-2915.1995.tb00132.x

[17] Schlein Y, Yuval B. Leishmaniasis in the Jordan Valley. Attraction of *Phlebotomus papatasi (Diptera*: *Psychodidae)* to plants in the field. J Med Entomol. 1987; 50:20–27. 10.1093/jmedent/24.1.87 3820245

[18] Schlein Y. Sandfly diet and *Leishmania*. Parasitol. 1986; 2:175–77. 10.1016/0169-4758(86)90150-x 15462814

[19] Durvasula R, Gumbs A, Panackal A, Kruglov O, Aksoy S, Merrifield RB, Richards FF, Beard CB. Prevention of insect-borne disease: an approach using transgenic symbiotic bacteria. Proc Natl Acad Sci USA. 1997; 94:3274–78. 9096383 10.1073/pnas.94.7.3274 PMC20359

[20] Berasategui A, Shukla S, Salem H, Kaltenpoth M. Potential applications of insect symbionts in biotechnology. Appl Microbiol Biotechnol. 2016; 100:1567–77. 10.1007/s00253-015-7186-9 26659224 PMC4737797

[21] Mukhopadhyay J, Braig HR, Rowton ED, Ghosh K. Naturally occurring culturable aerobic gut flora of adult *Phlebotomus papatasi*, vector of *Leishmania major* in the Old World. PLoS ONE. 2012; 7:e35748 10.1371/journal.pone.0035748 22629302 PMC3358311

[22] Hillesland H, Read A, Subhadra B, Hurwitz I, McKelvey R, Ghosh K, Das P, Durvasula R. Identification of aerobic gut bacteria from the Kala Azar vector, *Phlebotomus argentipes*: A platform for potential paratransgenic manipulation of sand flies. Am J Trop Med Hyg. 2008; 79:881–86. 19052297

[23] Hurwitz I, Hillesland H, Fieck A, Das P, Duvarsala R. The paratransgenic sand fly: a platform for control of *Leishmania* transmission. Parasites & Vectors. 2011;4: 82. 21595907 10.1186/1756-3305-4-82 PMC3121692

[24] Ben Ismail R. Incrimination de *Phlebotomus perniciosus* comme vecteur de *Leishmania infantum*. Arch Inst Pasteur de Tunis. 1993; 70:91–110.

[25] Chelbi I, Zhioua E. Biologiy of *Phlebotomus papatasi (Diptera*: *Psychodidae)* in the Laboratory. J Med Entomol. 2007; 44:597–00. 17695013 10.1603/0022-2585(2007)44[597:boppdp]2.0.co;2

[26] Croset H, Rioux JA, Master M, Bayar N. Les phlébotomes de la Tunisie (*Diptera*, *Phlebotominae*). Mise au point systématique, chronologique et éthologique. Ann Parasitol Hum Comp. 1978; 53:711–49. 754625

[27] Léger N, Pesson B, Madulo-Leblond G, Abonnenc E. Sur la différenciation des femelles du sous genre *Larroussisus* Nitzulescu, 1931(Diptera, Phlebotominae) de la région méditerranéenne. Ann Parasitol Hum Comp. 1983; 85:611–23. 6673646

[28] De Bruijn FJ. Handbook of molecular microbial ecology II: metagenomics in different habitats. Ed Wiley Blackwell 2011, pp: 96.

[29] Fisher MM, Triplett EW. Automated approach for ribosomal intergenic spacer analysis of microbial diversity and its application to freshwater bacterial communities. Appl Environ Microbiol. 1999; 65:4630–36 10508099 10.1128/aem.65.10.4630-4636.1999 PMC91617

[30] García-Martínez J, Acinas SG, Antón AI, Rodríguez-Valera F. Use of the 16S—23S ribosomal genes spacer region in studies of prokaryotic diversity. J Microbiol Meth. 1999; 36:55–64. 10.1016/s0167-7012(99)00011-1 10353800

[31] Daffonchio D, Cherif A, Brusetti L, Rizzi A, Mora D, Boudabous A, Borin S. Nature of polymorphisms in 16S-23S rRNA gene Intergenic Transcribed Spacer fingerprinting of *Bacillus* and related Genera. Appl Env Microbiol. 2000: 69:5128–37. 10.1128/AEM.69.9.5128-5137.2003 PMC194986 12957895

[32] Wheeler A, Oerther DB, Larsen N, Stahl DA, Raskin L. The oligonucleotide probe database. Appl Environ Microbiol. 1996; 62:3557*–*59. 8837410 10.1128/aem.62.10.3557-3559.1996 PMC168159

[33] Yu Z, Morrison M. Comparisons of different hypervariable regions of rrs genes for use in fingerprinting of microbial communities by PCR-denaturing gradient gelelectrophoresis. Appl Environ Microbiol. 2004; 70:4800–06. 10.1128/AEM.70.8.4800-4806.2004 15294817 PMC492348

[34] Sass AM, Sass H, Coolen MJ, Cypionka H, Overmann J. Microbial communities in the chemocline of a hypersaline deep-sea basin (Urania basin, Mediterranean Sea). Appl Environ Microbiol. 2001; 67:5392–402. 10.1128/AEM.67.12.5392-5402.2001 11722884 PMC93321

[35] Muyzer J, Smalla K. Application of denaturing gradient gel electrophoresis (DGGE) and temperature gradient gel electrophoresis (TGGE) in microbial ecology. Antonie van Leeuwenhoek. 1998; 73:127–41. 9602286 10.1023/a:1000669317571

[36] Ettoumi B, Bouhajja E, Borin S, Daffonchio D, Boudabous A, Cherif A. *Gammaproteobacteria* occurrence and microdiversity in Tyrrhenian Sea sediments as revealed by cultivation-dependent and -independent approaches. Syst Appl Microbiol. 2010; 33:222–31. 10.1016/j.syapm.2010.02.005 20413241

[37] Cole JR, Wang Q, Cardenas E, Fish J, Chai B, Farris RJ, Kulam-Syed-Mohideen AS, McGarrell DM, Marsh T, Garrity GM, Tiedje JM. The Ribosomal Database Project: improved alignments and new tools for rRNA analysis. Nucleic Acids Res. 2009; 37 (Database issue): D141–5. 10.1093/nar/gkn879 19004872 PMC2686447

[38] Stackerbrandt E, Goebel BM. Taxonomic Note: A Place for DNA-DNA Reassociation and 16S rRNA Sequence Analysis in the Present Species Definition in Bacteriology. Int J Syst Evol Microbiol. 1994; 44:846–49.

[39] Oksanen J, Blanchet FG, Kindt R, Legendre P, Minchin PR, O’Hara RB (2015). Vegan: Community Ecology. http://cran.r-project.org/package=vegan

[40] Sant'anna MRV, Darby AV, Brazil RP, Montoya-Lerma J, Dillon VM, Bates PA, Dillon RJ. Investigation of the bacterial communities associated with females of *Lutzomyia* sand fly species from South America. Plos one. 2012; 7:e42531 10.1371/journal.pone.0042531 22880020 PMC3411800

[41] Gouveia C, Asensi MD, Zahner V, Rangel EF, De Oliveira SMP. Study on the bacterial midgut microbiota associated to different Brazilian populations of *Lutzomyia longipalpis* (Lutz & Neiva) (*Diptera*: *Psychodidae*). Neotrop Entomol. 2008; 37:597–60. 19061048 10.1590/s1519-566x2008000500016

[42] McCarthy CB, Diambra LA, Pomar RVR. Metagenomic analysis of taxa associated with *Lutzomyia longipalpis*, vector of visceral leishmaniasis, using an unbiased high-throughput approach. PLoS Negl Trop Dis. 2011; 5:9. 10.1371/journal.pntd.0001304 PMC3167787 21909446

[43] Vivero RJ, Jaramillo NG, Cadavid-Restrepo G, Soto SI, Herrera CX. Structural differences in gut bacteria communities in developmental stages of natural populations of *Lutzomyia evansi* from Colombia's Caribbean coast. Parasit Vectors. 2016; 9:496 10.1186/s13071-016-1766-0 27618991 PMC5020466

[44] Volf P, Kiewegová A, Nemec A. Bacterial colonisation in the gut of *Phlebotomus duboscqi* (*Diptera*: *Psychodidae*): Transtadial passage and the role of female diet. Folia Parasitol. 2002; 49:73–7. 11993554 10.14411/fp.2002.014

[45] Akhoundi A, Bakhtiari R, Guillard T, Baghaei A, Tolouei R, Sereno D, Toubas D, Depaquit J, Abyaneh MR. Diversity of the bacterial and fungal microflora from the midgut and cuticle of Phlebotomine sand flies collected in North-Western Iran. PloS ONE. 2012; 7:11. 10.1371/journal.pone.0050259 PMC3511470 23226255

[46] Guernaoui S, Garcia D, Gazanion E, Ouhdouch Y, Boumezzough A, Pesson B, Fontenille D, Sereno D: Bacterial flora as indicated by PCR-temperature gradient gel electrophoresis (TGGE) of 16S rDNA gene fragments from isolated guts of phlebotomine sand flies (*Diptera*: *Psychodidae*). J Vec Ecol. 2011; 36:S144–7. 10.1111/j.1948-7134.2011.00124.x 21366767

[47] Maleki-Ravasan N, Oshaghi MA, Afshar D, Arandian MH, Hajibani S., Akhvan AA, Yakhckali B, Shirazi MH, Rassi Y, Jafari R, Aminian K, Fazeli-Varzaneh RA, Duvarsala R. Aerobic bacterial flora of biotic and abiotic compartments of a hyperendemic zoonotic cutaneous Leishmaniasis (ZCL) focus. Parasite & Vect. 8:63. 10.1186/s13071-014-0517-3 PMC4329651 25630498

[48] Mukhopadhyay J, Braig HR, Rowton ED, Ghosh K. Naturally occurring culturable aerobic gut flora of adult *Phlebotomus papatasi*, vector of *Leishmania major* in the Old World. PLoS ONE. 2012;7: 5. 10.1371/journal.pone.0035748 PMC3358311 22629302

[49] Li K, Chen H, Jiang J, Li X, Xu J, Ma Y. Diversity of bacteriome associated with *Phlebotomus chinensis* (Diptera: Psychodidae) sand flies in two wild populations from China. Sci Rep. 2016; 6:36406 10.1038/srep36406 27819272 PMC5098245

[50] Shannon P, Markiel A, Ozier O, Baliga NS, Wang JT, Ramage D, Amin N, Schwikowski B, Ideker T. Cytoscape: a software environment for integrated models of biomolecular interaction networks. Genome Res. 2003; 13:2498–2504. 10.1101/gr.1239303 14597658 PMC403769

[51] Matsumoto K, Izri A, Dumon H, Raoult D, Parola P. First detection of *Wolbachia spp*., including a new genotype, in sand flies collected in Marseille, France. J Med Entomol. 2008; 45:466–9. 18533441 10.1603/0022-2585(2008)45[466:fdowsi]2.0.co;2

[52] Minard G, Mavingui P, Moro CV. Diversity and function of bacterial microbiota in the mosquito holobiont. Parasit Vectors. 2013; 6:146 10.1186/1756-3305-6-146 23688194 PMC3667145

[53] Adler S, Theodor O. Attempts to transmit *Leishmania tropica* by bite: the transmission of *L*. *tropica* by *Phlebotomus sergenti*. Ann Trop Med Parasitol. 1929; 23:1–18.

[54] Dillon RJ, El Kordy E, Shehata M, Lane RP. The prevalence of a microbiota in the digestive tract of *Phlebotomus papatasi*. Ann Trop Med Parasitol. 1996; 90:669–73. 9039284 10.1080/00034983.1996.11813102

[55] Sant’Anna MRV, Diaz-Albiter H, Aguiar-Martins K, Al Salem WS, Cavalcante RR, Dillon VM, Bates PA, Genta FA, Dillon RJ: Colonization resistance in the sand fly gut: *Leishmania* protects *Lutzomyia longipalpis* from bacterial infection. Parasit & Vect. 2014; 7:329. 10.1186/1756-3305-7-329 PMC4112039 25051919

[56] Monteiro CC, Villegas LE, Campolina TB, Pires AC, Miranda JC, Pimenta PF, Secundino NF. Bacterial diversity of the American sand fly *Lutzomyia intermedia* using high-throughput metagenomic sequencing. Parasit Vectors. 2016; 9:480 10.1186/s13071-016-1767-z 27581188 PMC5007851

[57] Samie M, Wallbanks KR, Moore JS, Molineux DH. Glycosidase activity in the sandfly *Phlebotomus papatasi*. Comp Biochem Physiol. 1990; 96:577–79.

[58] Brook JS. *Stenotrophomonas maltophilia*: an emerging global opportunisitic pathogen. Clin Microbiol Rev. 2012; 25:2–41. 10.1128/CMR.00019-11 22232370 PMC3255966

[59] Bichaud L, Dachraoui K, Piorkowski G, Chelbi I, Moureau G, Cherni S, De Lamballerie X, Sakhria S, Charrel RN, Zhioua E. Isolation of Toscana virus from sand flies, Tunisia. Emerg Infect Dis. 2013;19: 322–324. 23460990 10.3201/eid1902.121463 PMC3559066

[60] Zoghlami Z, Chouihi E, Barhoumi W, Dachraoui K, Massoudi N, Ben Helel K, Habboul Z, Hadhri MH, Limam S, Mhadhbi M, Gharbi M, Zhioua E. Interaction between canine and human visceral leishmaniases in a holoendemic focus of Central Tunisia. Acta Trop. 2014; 139:32–8. 10.1016/j.actatropica.2014.06.012 25004438

[61] Barhoumi W, Fares W, Cherni S, Derbali M, Dachraoui K, Chelbi I, Ramalho-Ortigao M, Beier JC, Zhioua E. Changes of sand fly populations and *Leishmania infantum* infection rates in an irrigated village located in arid Central Tunisia. Inter J Envir Res Public Health. 2016;13: 329. 10.3390/ijerph13030329 PMC4808992 26999176

[62] Moraes CS, Seabra SH, Castro DP, Brazil RP, de Souza W, Garcia ES, Azambuja P: *Leishmania (Leishmania) chagasi* interactions with *Serratia marcescens*: ultrastructural studies, lysis and carbohydrate effects. Exp Parasitol. 2008; 118:561–568. 10.1016/j.exppara.2007.11.015 18206142

[63] Genes C, Baquero E, Echeverri F, Maya JD, Triana O. Mitochondrial dysfunction in *Trypanosoma cruzi*: the role of *Serratia marcescens* prodigiosin in the alternative treatment of Chagas disease. Parasites &Vect. 2011; 4:66. 10.1186/1756-3305-4-66 PMC3118961 21548954

[64] Kelly PH, Bahr SM, Serafim TD, Ajami NJ, Petrosino JF, Meneses C, Kirby JR, Valenzuela JG, Kamhawi S, Wilson ME. The Gut Microbiome of the Vector *Lutzomyia longipalpis* is Essential for Survival of *Leishmania infantum*. MBio. 2017; 8(1). 10.1128/mBio.01121-16 PMC5241394 28096483

[65] Teyssier C, Marchandin H, Jean-Pierre H., Diego I, Darbas H, Jeannot JL, Gouby A, Jumas-Bilak E. Molecular and phenotypic features for identification of the opportunistic pathogens *Ochrobactrum* spp. J Med Microbiol. 2005; 54:945 10.1099/jmm.0.46116-0 16157548

[66] Raffel TR, Register KB, Marks SA, Temple L. Prevalence of *Bordetella avium* infection in selected wild and domesticated birds in the Eastern USA. J Wild Dis. 2002; 38:40–6. 10.7589/0090-3558-38.1.40 11838227

[67] Botelho-Nevers E, Gouriet F, Lepidi H, Couvret A, Amphoux B, Dessi P, Raoult D. Chronic nasal infection caused by *Klebsiella rhinoscleromatis* or *Klebsiella ozaenae*: two forgotten infectious diseases. Inter J Infect Dis. 2007; 11:423–29. 10.1016/j.ijid.2006.10.005 17337224

[68] Bagley S. Habitat association of *Klebsiella* species. Infect Control. 1985; 6:52–8. 3882590 10.1017/s0195941700062603

[69] Finney CA, Kamhawi S, Wasmuth JD. Does the arthropod microbiota impact the establishment of vector-borne diseases in mammalian hosts? PLoS Pathog. 2015; 11:e1004646 10.1371/journal.ppat.1004646 25856431 PMC4391854

[70] Hiergeist A, Gläsner J, Reischl U, Gessner A. Analyses of Intestinal Microbiota: Culture versus Sequencing. ILAR J. 2015; 56:228–40. 10.1093/ilar/ilv017 26323632

[71] Janda JM, Abbott SL. 16S rRNA gene sequencing for bacterial identification in the diagnostic laboratory: pluses, perils, and pitfalls. J Clin Microbiol. 2007; 45:2761–2764. 10.1128/JCM.01228-07 17626177 PMC2045242

[72] Drancourt M, Bollet C, Carlioz A, Martelin R, Gayral JP, Raoult D. 16S ribosomal DNA sequence analysis of a large collection of environmental and clinical unidentifiable bacterial isolates. J Clin Microbiol. 2000; 3:3623–30. 10.1128/jcm.38.10.3623-3630.2000 PMC87447 11015374

[73] Yang B, Wang Y, Qian PY. Sensitivity and correlation of hypervariable regions in 16S rRNA genes in phylogenetic analysis. BMC Bioinformatics. 2016; 17:13. 27000765 10.1186/s12859-016-0992-y PMC4802574

